# PUS7-dependent Ψ reshapes specific synaptic gene exons to facilitate fear extinction memory formation

**DOI:** 10.1186/s13041-025-01250-6

**Published:** 2025-10-15

**Authors:** Runming Liu, Yuhan Dong, Zhipeng Gao, Jichun Shi, Ziyue Xu, Junhui Liu, Gaomeng Luo, Shengda Ye, Feiyang Zhang, Hongyu Xu, Xiang Li, Sha Liu, Wei Wei

**Affiliations:** 1 Brain Research Center, Zhongnan Hospital of Wuhan University, Wuhan, China; 2 Department of Neurosurgery, Zhongnan Hospital of Wuhan University, Wuhan, China; 3 Department of General Practice, Zhongnan Hospital of Wuhan University, Wuhan, China; 4 Frontier Science Center for Immunology and Metabolism, Wuhan University, Wuhan, China; 5 Medical Research Institute, Wuhan University, Wuhan, China

**Keywords:** RNA modification, Extinction, PTSD, PUS7

## Abstract

RNA modifications serve as dynamic regulators of neural plasticity through their ability to fine-tune transcript stability and splicing. Pseudouridine (Ψ), an evolutionarily conserved RNA modification catalyzed by pseudouridine synthases, plays established roles in neurodevelopment, yet its functional significance in activity-dependent behavioral adaptation remains poorly defined. Here, we investigate Ψ-mediated epitranscriptomic regulation within the infralimbic prefrontal cortex (ILPFC), a brain region requiring precise synaptic remodeling for the clinically relevant form of fear extinction memory. Combining transcriptome-wide pseudouridylation profiling with behavioral analysis in mice, we identified selective Ψ enrichment at exons of synaptic regulatory genes within ILPFC during fear extinction learning. Fear extinction in the ILPFC drives concomitant exonic Ψ deposition and upregulation of synaptogenic transcripts, processes that involve pseudouridine synthase PUS7. Crucially, PUS7 knockdown in the ILPFC selectively impaired fear extinction memory formation without altering baseline fear expression, establishing a causal link between Ψ-dependent RNA processing and activity-dependent synaptic structural remodeling in this microcircuit. Our findings demonstrate that PUS7-mediated Ψ modification spatiotemporally regulates activity-dependent RNA dynamics in the ILPFC, providing the evidence that epitranscriptomic mechanisms precisely coordinate synaptic gene expression within behaviorally defined brain sub-region. This work bridges molecular RNA biology with systems neuroscience, revealing a novel mechanism for activity-dependent regulation of fear extinction in ILPFC.

**Supplementary Information:**

The online version contains supplementary material available at 10.1186/s13041-025-01250-6.

## Introduction

Post-traumatic stress disorder (PTSD) is a debilitating neuropsychiatric disorder [1] characterized by complex psychopathological and neurobiological mechanisms, particularly synaptic dysfunction in key brain regions mediated by dysregulated molecular pathways [2, 3]. Elucidating the mechanisms underlying impaired fear extinction is critical for developing novel therapeutic strategies for PTSD [4].Fear extinction learning, an evolutionarily conserved and highly plastic process, is essential for survival by enabling organisms to adaptively respond to threatening or aversive stimuli [5]. In the classical Pavlovian fear conditioning paradigm, a widely utilized experimental model for studying extinction [6, 7]-repeated presentation of the conditioned stimulus (CS) in the absence of the unconditioned stimulus (US) drives the rapid reversal of learned threat associations [8]. Extinction-based exposure therapy, the first-line treatment for PTSD, leverages this mechanism to attenuate maladaptive fear responses through controlled re-exposure to trauma-related cues in a safe context [9]. Thus, deciphering the molecular underpinnings of fear extinction learning to enhance its efficacy represents a pivotal direction for improving PTSD therapeutics.

Similar to other forms of learning, the consolidation of long-term fear extinction memory relies on coordinated gene expression dynamics, with the infralimbic prefrontal cortex (ILPFC) serving as a critical hub for extinction circuitry [10]. Our work and others have demonstrated that this process involves diverse RNA-dependent mechanisms, including microRNA-mediated post-transcriptional regulation [11], long non-coding RNA (lncRNA)-guided local transcriptional modulation [12], and circular RNA (circRNA)-facilitated transcriptional priming [13]. Notably, RNA modifications, which fine-tune gene expression to regulate central nervous system (CNS) development and function [14], represent an underexplored layer of epigenetic regulation in fear-related plasticity. Pseudouridine (Ψ), the most abundant RNA modification, influences RNA stability, translation efficiency, and dynamic codon rewiring, with emerging links to neuropathology. For instance, elevated urinary Ψ levels have been reported in Alzheimer’s disease (AD) patients [15], though their direct correlation with AD symptomatology remains unclear. Furthermore, dysregulation of pseudouridine synthase (PUS) genes is closely associated with neuronal dysfunction: studies in murine embryonic neural systems reveal that Pus3 mRNA expression patterns suggest a role in neuronal development [16], while PUS3 deficiency in humans is linked to intellectual disability [17]. Similarly, PUS7 mutations are implicated in neurodevelopmental anomalies, including intellectual impairment, microcephaly, delayed language acquisition, and aggressive behaviors [18, 19]. Despite these advances, the functional relevance of Ψ in specific neuronal populations of the mammalian brain remains poorly characterized, and its role in regulating gene expression during learning and memory processes—particularly fear extinction—has yet to be elucidated. This knowledge gap underscores the urgent need to investigate Ψ-mediated epitranscriptomic mechanisms within fear extinction circuits, potentially unveiling novel therapeutic targets for PTSD.

## Materials and Methods

### Animals

Male C57BL/6 mice (10–12 weeks old) were group-housed (*n* = 4 per cage) under standardized laboratory conditions, including a 12-hour light/dark cycle (lights on 07:00–19:00) and ad libitum access to rodent chow and filtered water. Following a 7-day acclimatization period to stabilize circadian rhythms and minimize environmental stress, all behavioral and physiological assessments were conducted during the light phase under red-light illumination (λ > 650 nm) to accommodate murine nocturnal sensitivity. Experimental protocols were approved by the Animal Ethics Committee of Zhongnan Hospital, Wuhan University. Housing conditions, including ambient temperature (22 ± 1 °C) and relative humidity (50 ± 5%), were maintained in accordance with institutional guidelines for laboratory animal welfare.

### Tissue Processing and RNA Isolation

Tissue samples from the ILPFC of experimental mice were homogenized in 500 µL ice-cold phosphate-buffered saline (PBS; Gibco) using a pre-chilled Dounce tissue grinder. The homogenate was centrifuged at 300 × g for 5 min at 4 °C to pellet debris, and the supernatant was transferred to a fresh RNase-free tube. Total RNA was extracted from 100 µL of clarified homogenate using TRIzol™ reagent (Invitrogen) following the manufacturer’s protocol. Briefly, homogenate was mixed with 1 mL TRIzol, incubated for 5 min at room temperature, and phase-separated by adding 200 µL chloroform. After centrifugation (12,000 × g, 15 min, 4 °C), the aqueous phase was combined with 500µL isopropanol to precipitate RNA. The RNA pellet was washed twice with 75% ethanol, air-dried, and resuspended in 30µL RNase-free water.

### RNA Quantification and Quality Control

RNA concentration was determined using a Qubit™ RNA High Sensitivity (HS) Assay Kit on a Qubit 4.0 Fluorometer (Invitrogen). RNA integrity was verified by agarose gel electrophoresis (1.5% gel), with intact 18 S and 28 S ribosomal RNA bands confirming minimal degradation. All RNA samples were stored at − 80 °C until downstream analysis.

### Integrated LC–MS/MS Methodology for Ψ Modification Analysis

Total RNA was enzymatically hydrolyzed to ribonucleosides using benzonase (20 U), RNase T1 (10 U), and alkaline phosphatase (0.5 U) in 20mM ammonium acetate buffer (pH 5.3) containing 1 mM MgCl_2_ and 0.1 mM deferoxamine, followed by acetonitrile precipitation and lyophilization. Linear ion trap (LIT) and triple quadrupole (QQQ) scans were acquired on a triple quadrupole-linearion trap mass spectrometer (QTRAP), QTRAP^®^ 6500 + LC-MS/MS System, equipped with an ESI TurboIon-Spray interface, operating in positive ion mode and controlled by Analyst 1.6.3 software (Sciex). The ESI source operation parameters were as follows: ion source, ESI+; source temperature 550 ℃; ion sprayvoltage (IS) 5500 V; curtain gas (CUR) was set at 35 psi, respectively. RNA modifications were analyzed using scheduled multiple reaction monitoring (MRM). Data acquisitions were performed using Analyst 1.6.3 software (Sciex). Multiquant 3.0.3 software (Sciex) was used to quantify all metabolites. Mass spectrometer parameters including the declustering potentials (DP) and collision energies (CE) for individual MRM transitions were done with further DP and CE optimization. A specific set of MRM transitions were monitored for each period according to the metabolites eluted within this period.

### RNA dot blot analysis for Ψ modification

Total RNA (2 µg) was denatured in 2 × SSC buffer (300 mM NaCl, 30 mM sodium citrate, pH 7.0) containing 6.5% formaldehyde and 50% formamide at 65 °C for 15 min, then rapidly chilled on ice. Nitrocellulose membranes (Bio-Rad) were pre-equilibrated in 2× SSC for 10 min before sample spotting. Denatured RNA (2 µL per spot) was applied to the membrane using a vacuum dot blot apparatus (Bio-Rad), followed by UV crosslinking at 254 nm (150 mJ/cm²) for 1 h to immobilize RNA. incubated with anti-Ψ monoclonal antibody (1:500, MBL-d347-3) in blocking buffer overnight at 4 °C. After three 10-min washes with TBS-T (0.1% Tween-20), membranes were probed with AlexaFluor 680-conjugated goat anti-mouse IgG (1:10,000, LI-COR) for 1 h at 25 °C. Post-wash, signals were captured using an Odyssey^®^ Fc Imaging System (700 nm channel) and quantified with Image Studio™ Software (v5.2). Data were normalized to total RNA load (0.02% methylene blue staining) and validated against Ψ-depleted controls (PsiHyd-treated RNA).

### RT-qPCR

Complementary DNA (cDNA) was synthesized from 1 µg of total RNA using the HiScript II Reverse Transcriptase Kit (Vazyme Biotech Co., Ltd, Nanjing, China) following the manufacturer’s instructions. Quantitative PCR amplification was performed on a RotorGeneQ thermocycler (Qiagen, Germany) using ChamQ Universal SYBR qPCR Master Mix (Vazyme Biotech) and gene-specific primers (Supplementary Table 1). Phosphoglycerate kinase 1 (PGK1) was employed as an endogenous control to normalize variations in RNA input and reverse transcription efficiency. Reactions were conducted in duplicate for each sample, with the following cycling parameters: initial denaturation at 95 °C for 5 min, followed by 40 cycles of 95 °C for 10 s, 60 °C for 15 s, and 72 °C for 20 s. Relative transcript quantification was performed using the comparative ΔΔCq method, with beta-actin mRNA serving as the reference gene. To ensure reproducibility, all experiments included technical replicates and were independently repeated in at least two biological replicates.

### Ψ-RIP&Ψ-RIP-seq

RNA was isolated from the ILPFC in both RC and EXT groups, with biological replicates included (*n* = 3 per group). In our experiments, each biological replicate refers to an independently housed and treated individual animal. For each replicate, tissue from the ILPFC was collected and processed separately from a single mouse; no samples were pooled at any stage. All subsequent steps, including RNA extraction, library preparation, and sequencing (or other downstream assays), were performed independently for each animal. Thus, each n represents one independent animal.For the pseudouridine (Ψ) RNA immunoprecipitation (RIP), adapted from established protocols [20] with modifications. Briefly, 100ng of RNA per fraction was chemically fragmented into ~ 100-nucleotide segments using fragmentation buffer (10 mM ZnCl_2_, 10 mM Tris-HCl, pH 7.0) at 94 °C for 5 min, followed by quenching with 0.05 M EDTA. Input RNA aliquots were stored at − 80 °C for downstream normalization. For Ψ-specific enrichment, samples were incubated overnight at 4 °C with 5 µg of anti-Ψ polyclonal antibody (MBL) in IPP buffer (150 mM NaCl, 0.1% NP-40, 1 mM Tris-HCl, pH 7.4). Immune complexes were captured using Dynabeads Protein-G (Fisher Scientific) during a 2 h incubation at 4 °C. After stringent washing, bound RNA was eluted in 10 µl nuclease-free water, with 8 µl utilized for library preparation via the SMARTer Stranded Pico Input Mammalian Kit (Takara Bio), adhering to the manufacturer’s protocol. Libraries were purified using AMPure XP beads (Beckman Coulter) to exclude primer dimers and fragments exceeding 500 bp. Final libraries (size range: 200–500 bp, peak ~ 400 bp) were sequenced on an Illumina HiSeq 4000 platform using a single-flow cell conFiguration for paired-end reads. For data analysis, results from each biological replicate ( individual mouse) were analyzed separately and treated as independent data points. Statistical analyses were performed to compare groups based on these individual values, and no samples from different animals were pooled. All reported results reflect the biological variability among individual mice.

### Bioinformatic processing and Ψ modification analysis

Sequencing data preprocessing was performed using Cutadapt (v1.17) to eliminate adapter sequences at the 3′ terminus and trim low-quality bases (Phred score < 20). To deplete ribosomal RNA (rRNA) and PhiX control sequences, raw reads were aligned to their respective reference databases using Bowtie2 (v2.3.4.2), with unaligned reads retained for downstream analysis. High-quality reads were subsequently mapped to the mouse reference genome (GRCm39/mm39) using the STAR aligner (v2.7.10a) with default parameters, including splice junction-aware alignment to ensure comprehensive transcriptome coverage. Post-alignment processing involved the removal of PCR duplicates using SAMtools (v1.8; markdup -r), followed by filtering to retain high-confidence alignments (properly paired reads with mapping quality ≥ 20; flags -f2 -q 20). For Ψ modification analysis, differential pseudouridylation sites between the RC and EXT groups were identified using ExomePeak2 (v2.16.0), a computational framework optimized for Ψ-specific peak calling. Statistical significance of site-specific Ψ modifications was assessed using Przyborowski and Wilenski’s conditional binomial test (C-test) to compare Poisson-distributed read counts across experimental conditions. False discovery rates (FDR) were calculated using a sample-swapping permutation strategy [21]. Transcripts harboring significant Ψ modifications (FDR < 0.05) were subjected to GO enrichment analysis via DAVID Bioinformatics Resources (v6.8) to identify overrepresented biological processes, molecular functions, and cellular components. Genome-wide Ψ modification peaks were annotated and visualized using the Integrative Genomics Viewer (IGV, v2.7.2) to contextualize their genomic distribution and regulatory potential.

### Formaldehyde RNA Immunoprecipitation (fRIP) for PUS7 Antibody

The formaldehyde RNA immunoprecipitation (fRIP) assay was adapted to investigate RNA-protein interactions involving PUS7, following optimized protocols from [22, 23]. Briefly, samples were homogenized in ice-cold PBS and cross-linked with 0.1% methanol-free formaldehyde for 5 min at room temperature. Cross-linking was terminated by adding 125mM glycine, followed by centrifugation at 8,000 rpm for 2 min. Pelleted cells were washed three times with PBS supplemented with 1× protease inhibitor cocktail (PIC). For lysis, samples were resuspended in 1 mL of native lysis buffer (25 mM Tris-HCl pH 7.5, 150 mM KCl, 5 mM EDTA, 0.5% NP-40, 1:1000 RNaseOUT, 1 mMDTT, and 1× PIC). A 56 µL aliquot of each lysate was reserved as input control, while the remaining lysate was divided equally for immunoprecipitation (IP) with anti-PUS7 antibodies. For each IP reaction, 5 µg of anti-PUS7 antibody was added to the lysate and incubated with rotation at 4 °C for 2 h. Subsequently, 50 µL of pre-washed Dynabeads Protein G (Fisher Scientific) was added to the antibody-lysate mixture and rotated for an additional 1 h at 4 °C to capture antibody-RNA-protein complexes.Beads were washed three times with native lysis buffer to remove nonspecific interactions and resuspended in 56 µL of ultrapure water. Reverse cross-linking was performed by adding 33 µL of reverse-crosslinking buffer (3 × PBS, 6% N-lauroylsarcosine, 30 mM EDTA, 15 mM DTT, 1:1000 RNaseOUT, and 1 × PIC) and incubating at 42 °C for 1 h, followed by 55 °C for 1 h to dissociate RNA-protein complexes. RNA was purified using RNAClean XP beads (Beckman Coulter) and treated with DNase I (Zymo Research) according to the manufacturer’s protocol to eliminate genomic DNA contamination. Purified RNA was eluted in 11 µL of RNase-free water and analyzed via qR-PCR to quantify target RNA enrichment.

### PUS7 knockdown constructs

Lentiviral plasmids for PUS7 knockdown were generated by inserting PUS7-specific shRNA or scrambled control sequences (Supplementary Table 1) immediately downstream of the H1 promoter in a modified FG12 vector (FG12H1, derived from the FG12 vector originally provided by D. Baltimore, CalTech), as previously described. Lentivirus production and handling followed protocols approved by the and the WuHan University.

### Behavioral tests

Fear conditioning experiments were conducted in two distinct contexts (Context A: steel grid floor with vinegar odorant; Context B: white plastic-covered floor with almond odorant and LED illumination) using Coulbourn Instruments chambers. Mice underwent fear conditioning in Context A with three pairings of a 120 s, 80 dB, 16 kHz tone (CS) co-terminating with a 0.7 mA foot shock (US), followed by exclusion of subjects showing < 30% freezing during the final CS. For extinction training in Context B, mice received 30 non-reinforced 120 s CS presentations (5 s intervals) after a 120 s acclimatization. Context-exposure controls were exposed to equivalent durations of A/B without stimuli. Retention tests assessed freezing during three CS presentations in Context B after 24 h, quantified via FreezeFrame software. Animals with off-target injections or insufficient gene knockdown (< 70% efficiency) were excluded, and experimenters remained blinded to conditions throughout analysis.

### Open-field test

Mice of both types (Control and shPUS7) were randomly selected for open field. Mice were subjected to an open-field test to evaluate generalized anxiety or reduced spontaneous locomotor activity. The test was performed in a soundproof room with dim white lighting (60 ± 3 lx). Each mouse was placed at the center of a white plastic open-field apparatus (30 × 30 × 30 cm) and its movements were recorded by an overhead camera for 25 min. The videos were analyzed using the Any-Maze animal tracking software (Xinruan Information Technology Co. Ltd., Shanghai, China) to measure parameters such as total distance traveled, frequency of entries into the central region, and the cumulative time spent in the center zone, defined as a 15 × 15 cm square at the center of the arena. Mice subjected to the open field test were not further analyzed for genetic testing.

### Behavioral training for tissue collection

Naive mice remained undisturbed in their home cages until euthanasia. Fear-conditioned (FC) mice received three pairings of a 120 s, 80 dB, 16 kHz pure tone (conditioned stimulus, CS) co-terminating with a 1 s, 0.7 mA foot shock (unconditioned stimulus, US) in Context A (120 s intertrial interval, ITI). One day later, FC mice were transferred to Context B: the EXT group received 30 non-reinforced 120 s CS presentations (5 s ITI), whereas the RC group remained in Context B for an equivalent duration without CS exposure. Animals showing significant fear reduction during training were excluded. Tissues were collected immediately after Context B exposure (RC) or extinction (EXT), with additional exclusion criteria applied for off-target injections or ineffective gene knockdown.

### Western blot analysis

Proteins were extracted using RIPA lysis buffer (servicebio) supplemented with protease/phosphatase inhibitors. Lysates were centrifuged at 12,000 × g for 15 min at 4 °C, and protein concentrations were quantified via BCA assay. Samples (20–40 µg) were denatured in Laemmli buffer, resolved on 8–12% SDS-PAGE gels, and transferred to 0.45 μm PVDF membranes (Merck) using the GenScript FastBlot system (17 min transfer). Membranes were blocked with 5% non-fat milk, incubated overnight at 4 °C with primary antibodies, and probed with HRP-conjugated secondary antibodies (1 h, room temperature). Signals were developed using chemiluminescent substrate (Thermo Fisher) and imaged on a ChemiDoc system (Bio-Rad). GAPDH served as loading controls.

### Dendritic spine analysis

For in vivo dendritic spine analysis, neurons within the ILPFC were sparsely labeled via stereotaxic viral injection. Sparse labeling was achieved using a commercial recombinant adeno-associated virus (rAAV) (BC-SL002, Bulinkesi, Shenzhen, China) expressing the red fluorescent protein mCherry. A total of 100 ng rAAV was injected per side into the ILPFC using standard stereotaxic procedures (as detailed in the previous brain stereotaxic injection methods section). Following co-injection of either PUS7 targeting shRNA or control shRNA into the same ILPFC region and an appropriate survival period, animals were perfused and brains sectioned. Fluorescently labeled ILPFC neurons within 20 μm thick tissue sections were imaged using a confocal microscope equipped with a 63× oil-immersion objective and Airyscan. Dendritic spines on these directly sparsely labeled neurons were subsequently quantified manually using ImageJ software.

## Results

### The detection of pseudouridine modification within ILPFC during fear extinction

**Fig. 1:**
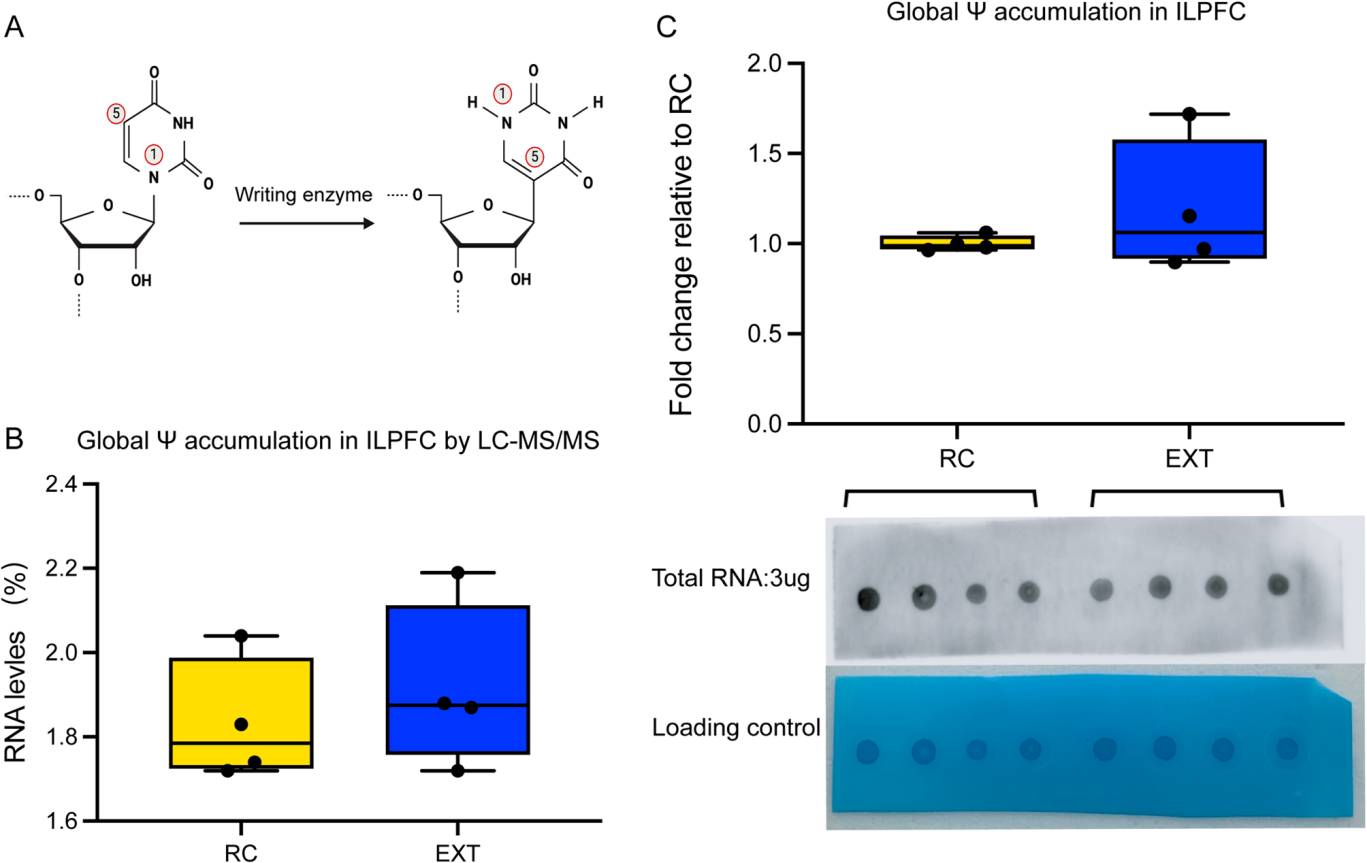
Detection of global Pseudouridine (Ψ) levels. **A** A schematic diagram of the transformation of uracil to Ψ. **B** Global Ψ level demonstrates an increased trend by extinction training (EXT) detected by LC-MS /MS. **C** Dot blot assay shows global accumulation of Ψ

Ψ, the most abundant RNA modification across all domains of life, exhibits significant abundance in mammalian mRNAs with Ψ/U ratios of 0.2–0.6% in human cells and murine tissues [24]. This highly conserved modification arises through base-specific isomerization involving 180° rotation of the uridine base [25] (Fig. 1A) and plays essential roles in RNA function. In ILPFC, a critical hub for fear extinction memory [26, 27], global LC-MS/MS quantification revealed an increased trend but no significant change of Ψ levels (constituting 1–2% of nucleosides) following extinction training (Fig. 1B). Consistently, Ψ-specific dot blot analysis confirmed this observation in Ψ modification levels post-extinction (Fig. 1C). A substantial enrichment of Ψ modification was observed in the ILPFC region., Given the notably higher Ψ abundance observed in the ILPFC (1–2%) relative to other regions (0.2–0.6%), it is reasonable to hypothesize that Ψ modification may play a more prominent role in the function of the ILPFC. (Supplementary Table 2). Collectively, these findings suggest that Ψ may function as a regionally restricted epitranscriptomic marker exhibiting dynamic regulation during fear extinction. Its locus-specific accumulation may fine-tune gene expression programs underlying memory reorganization.

### The dynamic Ψ distribution in the ILPFC in response to extinction learning

**Fig. 2:**
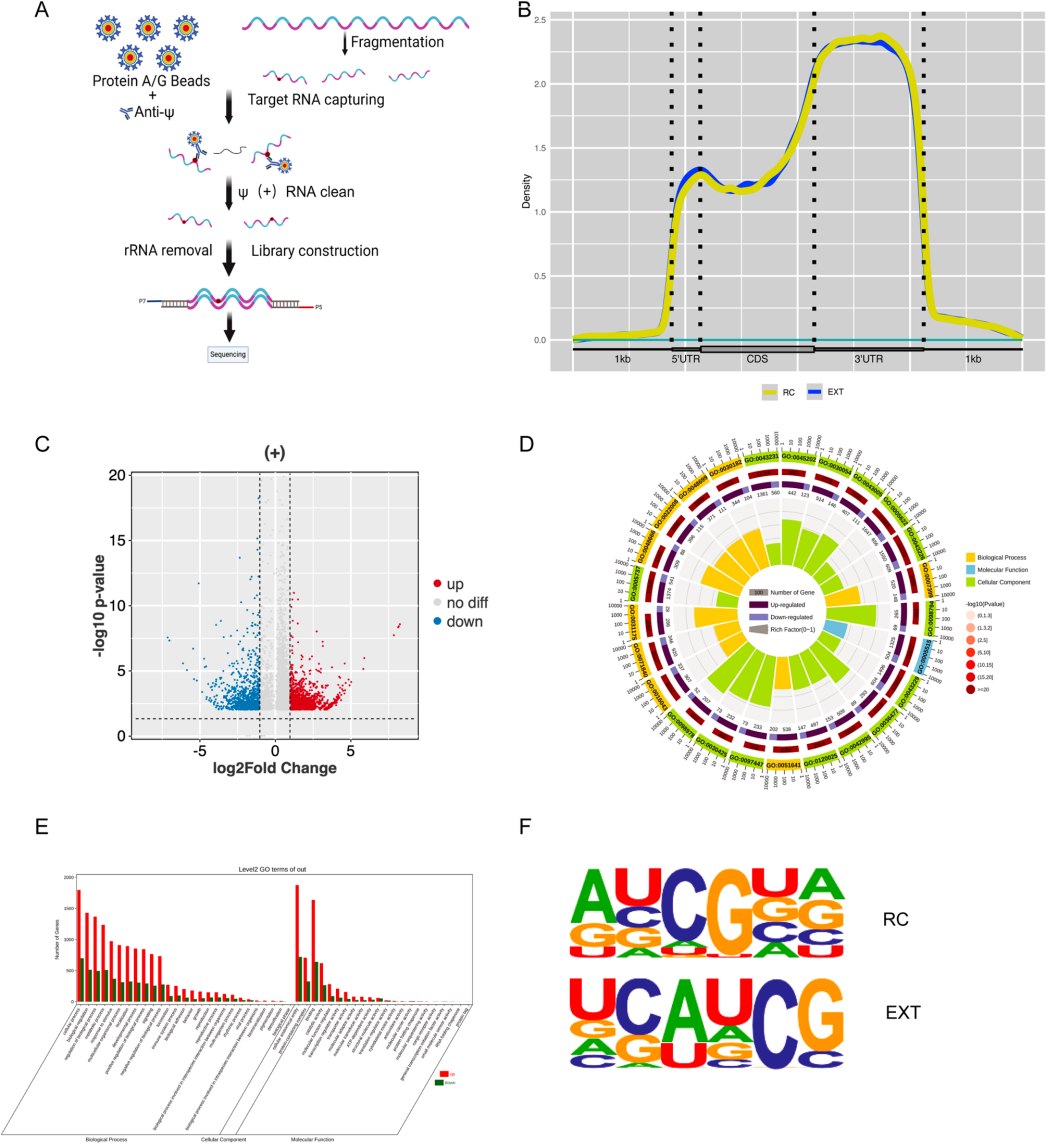
Ψ-RIP-seq characterized the distribution of Ψ modifications along RNA transcripts within the ILPFC following fear extinction. **A** Schematic Representation of Ψ-rip-seq Technology. **B** The Guitar plot shows that the Ψ deposition is mainly located in the 3’UTR region and the CDS region. **C** Volcano plot shows the fold change against the p value, blue and red dots represent genes with significant change (*p* < 0.05, FC > 1.0). **D** Circular plot showing GO enrichment analysis results, categorized into Biological Process (yellow), Molecular Function (blue), and Cellular Component (purple). The inner ring indicates the number of genes, with up-regulated (light green) and down-regulated (light red) genes. The middle ring shows the rich factor (light pink to dark red), and the outer ring displays -log10(P-value) (light gray to black). This visualization highlights significantly enriched GO terms and their biological functions. **E** Bar chart showing the number of upregulated (red) and downregulated (green) genes across Level 2 Gene Ontology (GO) terms after extinction treatment. The x-axis categorizes terms into Biological Process, Cellular Component, and Molecular Function. Key changes are observed in “cellular process,” “biological regulation,” and “binding,” indicating significant functional shifts post-treatment. **F** Ψ motif within RC and EXT groups

To identify specific Ψ modification sites, we performed transcriptome-wide mapping of Ψ modifications in ILPFC using Ψ-specific immunoprecipitation sequencing (Ψ-RIP-Seq) post fear extinction (Fig. 2A). Extinction training (EXT) group and retention control (RC) groups (n = 3 biological replicates/group) demonstrated conserved spatial patterning, with Ψ modifications predominantly enriched in 3’ untranslated regions (3’UTRs) and significantly represented in coding sequences (CDS) and 5’UTRs (Fig. 2B). Genome-wide profiling identified 6,069 pseudouridylated sites (Supplementary Table 3). Differential analysis revealed extinction-induced Ψ remodeling at specific loci: 963 sites (15.9%) exhibited increased modification occupancy while 419 sites (6.9%) showed decreased occupancy (Fig. 2C); Gene Ontology (GO) analysis revealed bidirectional modulation of major pathways under extinction (Fig. 2D). Specifically, transcripts exhibiting significantly elevated Ψ occupancy demonstrated pronounced enrichment across major GO categories. This enrichment was particularly evident in Molecular Function terms, including binding and catalytic activity, and in Biological Process terms, such as cellular process, biological regulation, regulation of biological process, and metabolic process (Fig. 2E). De novo motif analysis confirmed evolutionary conservation of the “AUCG” recognition sequence within Ψ peaks (Fig. 2F), supporting targeted pseudouridylation mechanisms.

### The Ψ synthase enzyme PUS7 is necessary for fear extinction memory formation

**Fig. 3:**
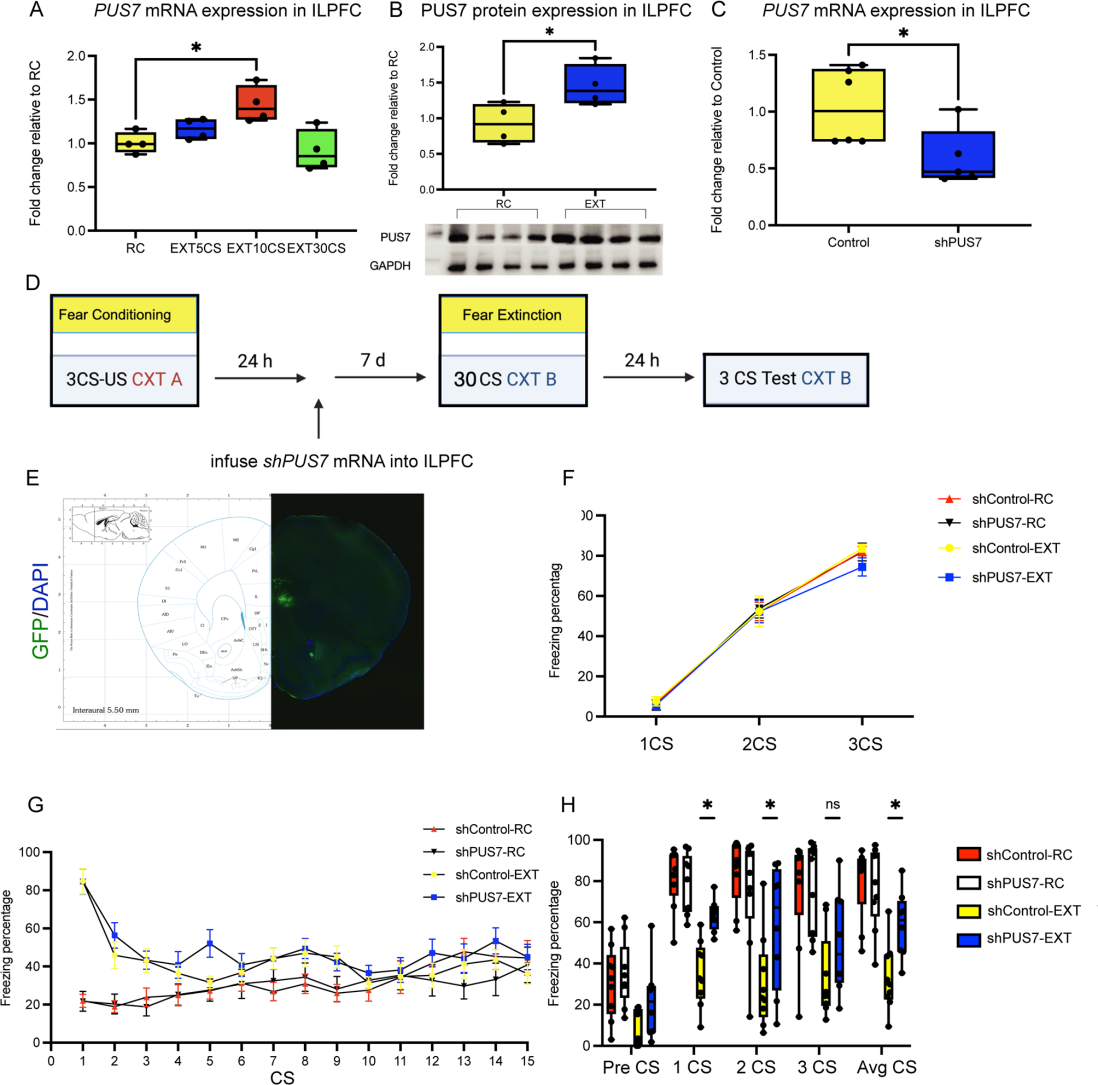
PUS7 activity is necessary for fear extinction memory formation. **A** Learning-induced PUS7 mRNA expression in ILPFC after extinction. EXT 10CS shows significantly higher expression compared to RC (*n* = 4 biologically independent animals in each group, one-way ANOVA, * *p* < 0.05). Data are presented as box plots. **B** The expression of PUS7 protein in ILPFC after extinction. Compared with RC, the expression level of EXT was significantly increased (*n* = 4 biologically independent animals in each group, double-tailed unpaired Student’s t-test, *** *p* < 0.001). The data is represented by a bar chart. **C** RT-qPCR showed that after injection of PUS7 shRNA, PUS7 mRNA in ILPFC was significantly downregulated (*n* = 5–6 biologically independent animals in each group, double-tailed unpaired Student’s t-test, * *p* < 0.05). **D** Schematic of the behavioral protocol used to test the effect of lentiviral mediated knockdown of PUS7 in the ILPFC on fear extinction memory. CTX, context; CS, conditioned stimulus; US, unconditioned stimulus. **E** Representative images of ILPFC infected by PUS7 shRNA lentivirus; Green: GFP, Blue: DAPI. **F** All experimental groups were subjected to identical fear conditioning protocols, and subsequent quantitative assessment revealed no significant differences in freezing behavior between the groups. **G**There were no significant differences between the PUS7 shRNA and control groups during fear acquisition, and no effect of PUS7 shRNA on performance during within-session extinction training. (H) PUS7 knockdown led to a significant impairment in memory for fear extinction in AvgCS (*n* = 8–9 biologically independent animals, two-way ANOVA, F(4,155) = 26.93, ****p < 0.0001, Sı ´da ´ k’s post hoc analysis, 1CS: shControl EXT versus shPUS7EXT, *p < 0.05; 2CS: shControl EXT versus shPUS7EXT, *p < 0.05; 3CS: shControl EXT versus shPUS7EXT, *p* > 0.05;AvgCS: shControl EXT versus shPUS7EXT, *p < 0.05). CS, conditioned stimulus; preCS, a 2 min acclimation pretest period to minimize context generalization 2CS; 1CS: 1 toneCS exposures; 2CS: 2 toneCS exposures; 3CS: 3 toneCS exposures; AvgCS; 3 toneCS exposures average of 3 toneCS exposures

The extensive dynamic distribution of pseuduridine levels in mRNA can be regulated [28], the pseudouridine synthase enzyme family, including specific pseudouridine synthases (PUS) and Dlkc1, functions as established writers of Ψ modifications in RNA. To investigate the role of these enzymes in fear extinction, we profiled the expression of key PUS family members-Pus1, Trub1, Rpusd3, Trub2, Rpusd4, Dlkc1and PUS7-in the ILPFC under extinction conditions. Experimental groups were exposed to varying numbers of conditioned stimuli (CS) during extinction training (5 CS, 10 CS, or 30 CS) process, with subsequent quantitative PCR (qPCR) analysis performed to quantify mRNA levels. qPCR analysis revealed a significant, time-dependent upregulation of PUS7 mRNA in extinction-trained mice compared to control, reaching statistical significance specifically in the 10 CS EXT group (One-way ANOVA, *p* < 0.05; Fig. 3A). In contrast, no significant changes in mRNA expression were detected for Pus1, Trub1, rPusd3, Trub2, rPusd4 or Dlkc1 across the extinction groups (Fig. S1A-F). Subsequently, Western blot analysis confirmed the elevated expression of the PUS7 protein post extinction training (Fig. 3B). This observation suggests a potential regulatory role for PUS7 in neurobiological processes associated with fear extinction. To address the functional role of PUS7, we employed lentivirus-mediated PUS7 shRNA to selectively knock down PUS7 expression in the ILPFC, a brain region critically implicated in fear extinction memory. Subsequent behavioral paradigms, including extinction training and testing, were conducted to systematically assess the impact of PUS7 suppression on fear extinction dynamics (Fig. 3D). Virally transduced ILPFC tissue exhibited high transfection efficiency and a significant reduction in PUS7 expression (Fig. 3C and E). Behavioral tests were subsequently conducted following the paradigm in Fig. 3D. Firstly, we confirmed that mice in all groups received equivalent fear training prior to virus injection and extinction training (Fig. 3F). PUS7 shRNA had no effect on within-session performance during extinction training (Fig. 3G), nor did it affect fear expression in mice subjected to fear conditioning and tested in a novel context prior to extinction training (Fig. 3H, left). However, mice receiving PUS7 shRNA showed significant impairment in fear extinction memory (Fig. 3H, right), with no effect on anxiety-like behavior in the open-field test (Fig. S2A–C). These findings establish that PUS7 specifically regulates fear extinction memory. However, whether this regulation occurs through modulation of Ψ levels remains unresolved. To address this mechanistic gap, we performed the following experiments.

### PUS7 mediated accumulation of Ψ facilitates the fear extinction memory formation

**Fig. 4:**
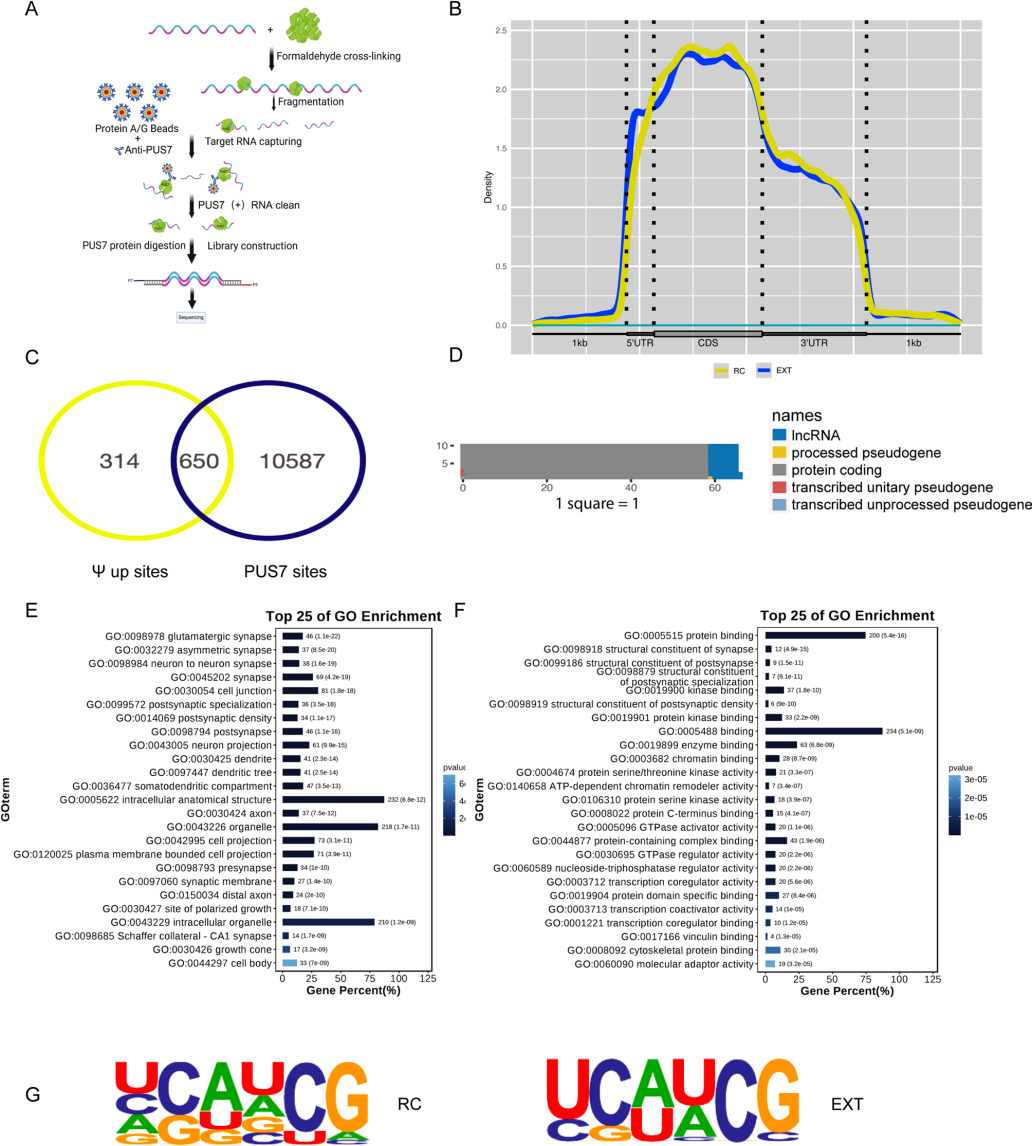
PUS7-mediated increase in Ψ modification regulates synaptic learning and memory in ILPFC during fear extinction. **A** Schematic representation of the PUS7-fRIP-seq technology. **B** The Guitar plot indicates that the signal intensity is predominantly concentrated in the CDS and 3’UTR regions, with lower levels observed in the 5’UTR and 1 kb upstream/downstream regions. **C** The Venn plot shows the overlap between the extinction upregulated pseudouracil (Ψ) sites (314 sites, yellow) and the target sites (10,587 sites, blue) bound to PUS7 in the extinction group. The intersection (650 loci) indicates the cooperative role of Ψ modification and PUS7 in post-transcriptional regulation during the process of extinction. **D** 650 pseudouracil modification sites mediated by PUS7.The proteins coding (gray) dominates, followed by IncRNA(blue), and other types are the smallest in quantity. **E** Top 25 GO terms enriched in synaptic function genes. The bar chart shows gene percentages and p-values, highlighting key synaptic terms like glutamatergic synapse (GO:0098978) and asymmetric synapse (GO:0032279), emphasizing their critical roles in synaptic processes. **F** The top 25 enriched Gene Ontology (GO) terms, with bars representing gene percentages and color intensity indicating p-values. Key synaptic-related terms include “structural constituent of synapse” (GO:0098918) and “structural constituent of postsynapse” (GO:0099186), emphasizing their critical roles in synaptic function. **G** Motifs of PUS7 binding sites in the mouse neuronal genome identified by PUS7-fRIP-seq

Accumulating evidence implicates Ψ modifications, catalyzed by pseudouridine synthases such as PUS7, as critical regulators of neurobiological adaptations. To directly test whether PUS7 mediates Ψ modification during fear extinction learning, we assessed the overlapping of PUS7-bound RNA in the ILPFC of EXT-trained mice using formaldehyde RNA immunoprecipitation sequencing (fRIP-seq) with Ψ-modified RNA peaks (Fig. 4A). Genome-wide mapping of PUS7 binding revealed broad occupancy, predominantly within coding sequences (CDS) (Fig. 4B). We identified 650 overlapping sites where PUS7 binding coincided with positions exhibiting increased Ψ modification specifically in the EXT group (Fig. 4C), strongly indicating that PUS7 directly catalyzes site-specific pseudouridylation in this context (Supplementary Table 4). Critically, statistical analysis confirmed that the majority of these 650 PUS7-catalyzed Ψ sites resided on mRNA molecules under extinction conditions (Fig. 4D). To investigate the functional significance of these specific mRNA modification sites, we performed Gene Ontology (GO) analysis, which demonstrated significant enrichment for synaptic pathways. Specifically, these mRNAs were enriched for glutamatergic synapse organization and structural plasticity mechanisms essential for learning and memory (Fig. 4E-F), including key terms such as postsynaptic density components and neuron-to-neuron synaptic signaling, highlighting their potential role in activity-dependent synaptic remodeling. Furthermore, motif analysis of PUS7-bound RNA identified a conserved “UCAUCG” sequence present in both RC and EXT groups (Fig. 4G). Collectively, these findings demonstrate that extinction training induces PUS7 upregulation and establish PUS7 as a pivotal post-transcriptional regulator catalyzing Ψ modifications within the adult brain. This RNA modification driven mechanism may contribute to molecular reprogramming associated with adaptive behavioral responses and fear extinction memory. These findings are in line with previous studies showing that epigenetic molecular mechanisms are involved in regulating synaptic functions underlying memory formation.

### Concurrent elevation of exonic pseudouridine modifications and synaptogenic gene expression in fear extinction

**Fig. 5:**
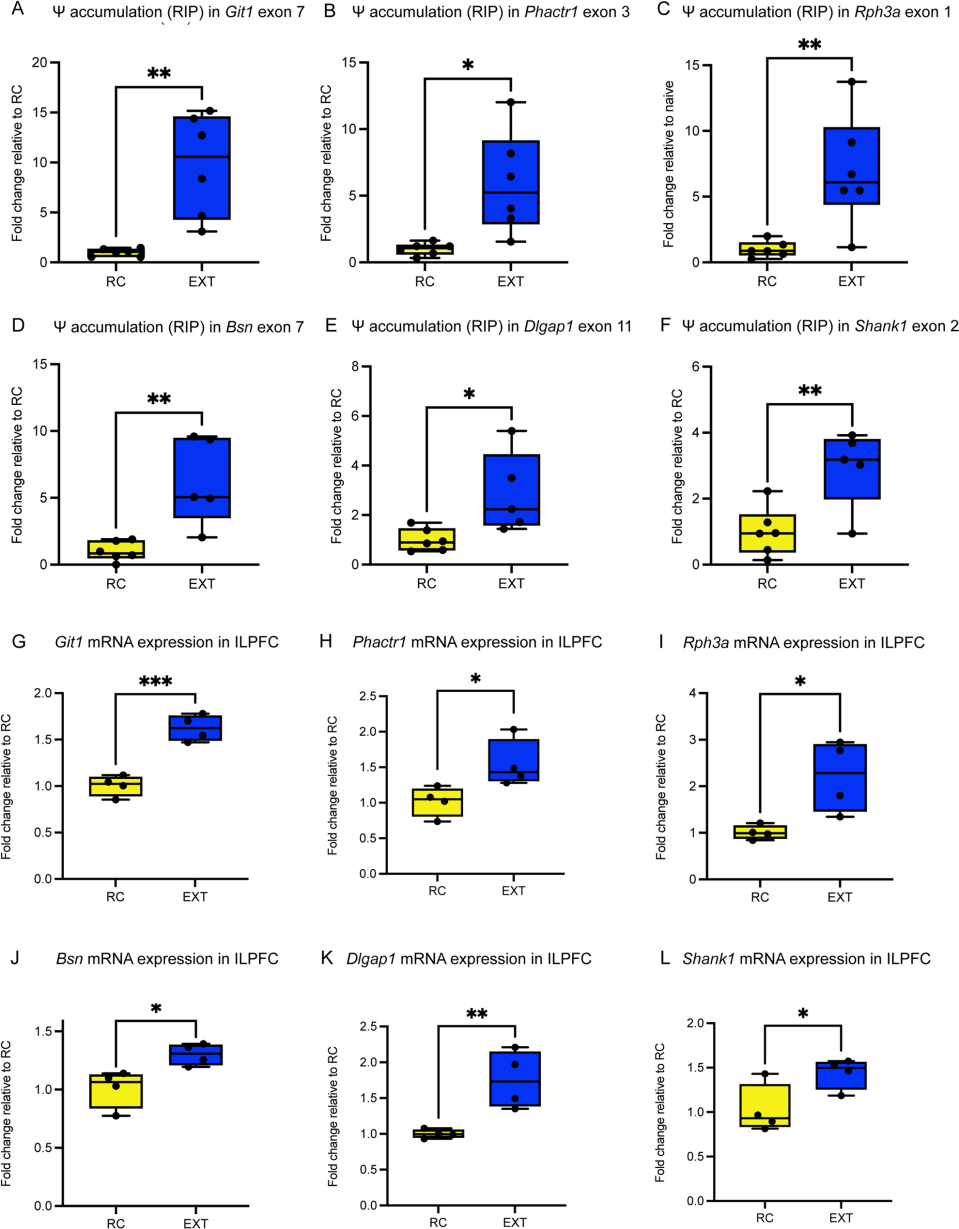
PUS7-mediated Ψ accumulation in synaptic-related genes was associated with activated gene expression. **A–F**The alterations in Ψ modification levels following extinction training were confirmed through Ψ-RIP-qPCR analysis. **A** Git1, **B** Phactr1, **C** Rph3a, **D** Bsn, **E** Dlgap1, and **F** Shank1 (*n* = 5–6 biological replicates per group, two-tailed unpaired Student’s t test). Error bars represent SEM; * *p* < 0.05, ** *p* < 0.01. Altered mRNA expression of synapse-associated genes following EXT, quantified by RT-qPCR(**G** Git1, **H** Phactr1, **I** Rph3a, **J** Bsn, **K** Dlgap1, and **L** Shank1(*n* = 4 biological replicates per group, two-tailed unpaired Student’s t test). Error bars represent SEM; * *p* < 0.05, ** *p* < 0.01,, *** *p* < 0.001

Gene Ontology (GO) enrichment analysis of Ψ-modified transcripts demonstrated significant enrichment of synaptic pathway-related genes. To investigate dynamic changes in Ψ modification during extinction, we selected synaptic function-associated sites from 650 overlapping Ψ-modified loci for validation. These sites were analyzed via Ψ-specific RNA immunoprecipitation quantitative PCR (Ψ-RIP-qPCR). In a fear extinction mouse model, anti-Ψ RIP-qPCR confirmed elevated Ψ modification levels at exonic sites of key synaptic genes (Git1, Phactr1, Rph3a, Bsn, Dlgap1 and Shank1) (t-test, *p* < 0.05; Fig. 5A–F). The upregulation of mRNA levels for these synaptogenic genes in the extinction group aligns with the observed increase in exonic pseudouridylation (t-test, *p* < 0.05; Fig. 5G–L). This concordance supports a model wherein cumulative Ψ modifications promote transcript stability, leading to increased mRNA abundance during extinction learning. To further investigate whether this process was mediated by PUS7, we injected a PUS7-targeting shRNA virus into the ILPFC brain region, followed by extinction training. Remarkably, compared to the control, mRNA that previously showed a cumulative increase in exon pseudouridine modifications in the EXT group exhibited no significant difference after PUS7 knockdown (t-test, *p* > 0.05; Fig S3A–F). These results indicate that PUS7-mediated accumulation of pseudouridine is essential for regulating the expression of these synaptic genes at the mRNA level.

#### Effects of shPUS7 Injection on Pseudouridine Deposition and Dendritic Spine Morphology

**Fig. 6:**
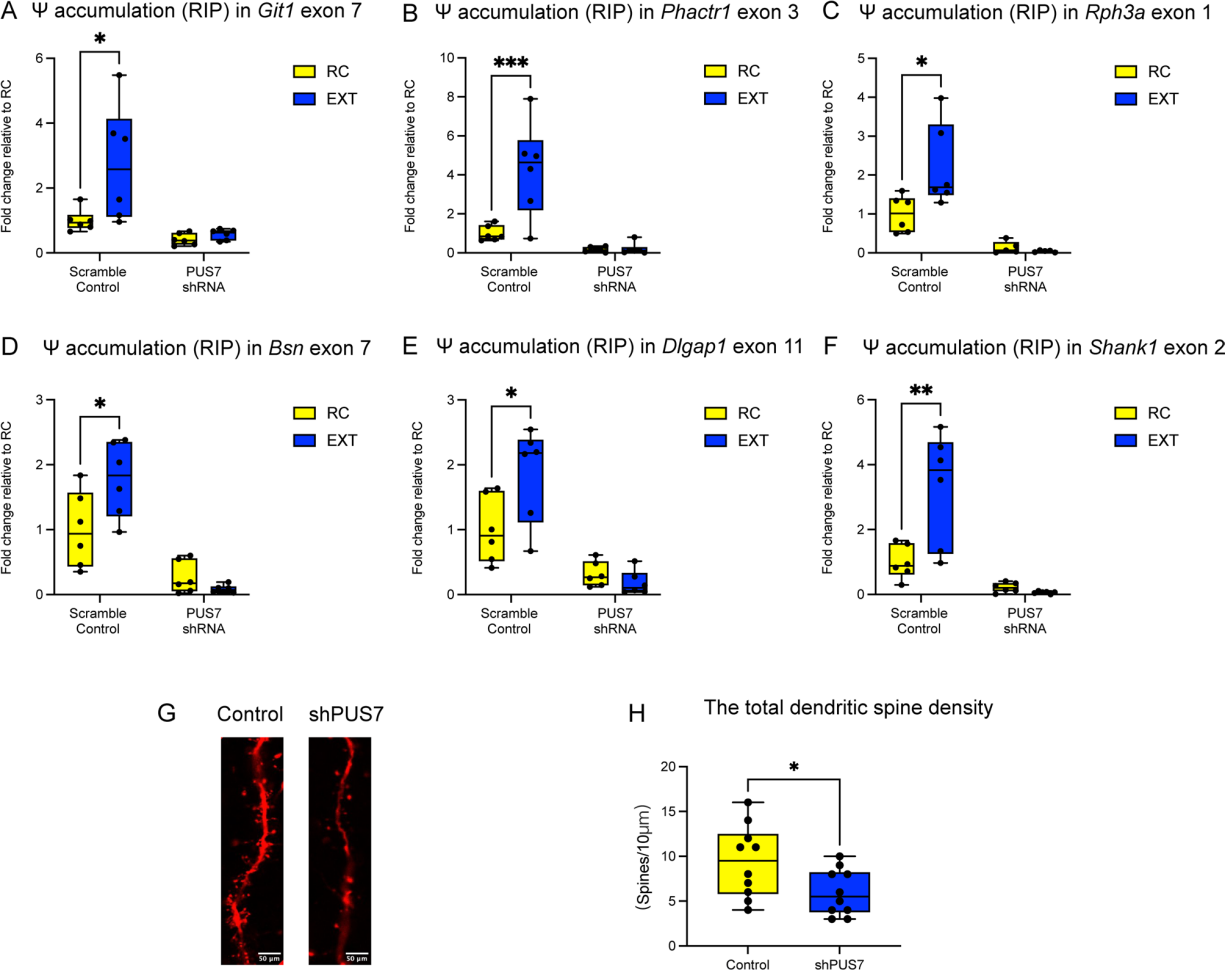
PUS7 knockdown impairs pseudouridine deposition and dendritic spine density. **A** PUS7 shRNA blocked the learning-induced increase in Ψ occupancy in Git1 exon7 (*n* = 6 biologically independent animals per group, two-way ANOVA, “F (1, 20) = 4.523, *p = 0.0461”; Dunnett’s post hoc test: scrambled control RC versus scrambled control EXT, *p < 0.05, **B** The deposition of Ψ occupancy in Phactr1 exon3(*n* = 6 biologically independent animals per group, two-way ANOVA, “F (1, 20) = 10.27, *p = 0.0045”; Dunnett’s post hoc test: scrambled control RC versus scrambled control EXT, ****p < 0.0001. **C** The deposition of Ψ occupancy in Rph3a exon1(*n* = 6 biologically independent animals per group, two-way ANOVA,“F (1, 18) = 6.030, *p = 0.0245”; Dunnett’s post hoc test: scrambled control RC versus scrambled control EXT, *p < 0.05). **D** The deposition of Ψ occupancy in Bsn exon7(*n* = 6 biologically independent animals per group, two-way ANOVA, “F (1, 20) = 7.256, *p = 0.0140”; Dunnett’s post hoc test: scrambled control RC versus scrambled control EXT, *p < 0.05). **E** The deposition of Ψ occupancy in Dlgap1 exon11(*n* = 6 biologically independent animals per group, two-way ANOVA, “F (1, 20) = 6.825, *p = 0.0167”; Dunnett’s post hoc test: scrambled control RC versus scrambled control EXT, *p < 0.05). **F** The deposition of Ψ occupancy in Shank1 exon2(*n* = 6 biologically independent animals per group, two-way ANOVA, “F (1, 20) = 10.75, **p = 0.0038”; Dunnett’s post hoc test: scrambled control RC versus scrambled control EXT, **p < 0.01). **G** Representative micrographs of dendrites labeled using the sparse neuron labeling method. Scale bar: 5 μm. **H** Bilateral microinjection of PUS7 shRNA into the infralimbic prefrontal cortex (ILPFC) significantly reduced dendritic spine density compared to control (unpaired t-test; * *p* < 0.05; *n* = 10 biologically independent animals per group)

We investigated whether extinction learning induces increased Ψ modification at specific exonic sites of synaptic genes, and whether this process depends on PUS7. To address this, we performed RNA immunoprecipitation to measure Ψ levels at selected exons of several key synapse-related genes. In the scramble control group, EXT produced a significant increase in Ψ deposition at these exonic sites compared to the RC group. However, when PUS7 expression was knocked down using shRNA, this learning-induced increase in Ψ levels was completely abolished. In the PUS7 shRNA group, Ψ modification levels did not differ significantly between extinction and control conditions in any of the genes examined, including Git1 exon 7 (Fig. 6A), Phactr1 exon 3 (Fig. 6B), Rph3a exon 1 (Fig. 6C), Bsn exon 7 (Fig. 6D), Dlgap1 exon 11 (Fig. 6E), and Shank1 exon 2 (Fig. 6F). Together, these findings demonstrate that PUS7 is required for the experience-dependent accumulation of pseudouridine at specific exons of synaptic genes during extinction learning, suggesting an essential role for PUS7-mediated pseudouridylation in the epigenetic regulation of memory processes.Given that the overlapping genes where PUS7 binding coincided with augmented Ψ modification are enrich in synaptic pathway (Fig. 4E), and the established links between synaptic structural plasticity and memory processes [29, 30], we hypothesized that PUS7 modulates dendritic spine remodeling in synapstic gene. To test this, sparsely labeled viruses were injected into the ILPFC to examine PUS7 knockdown effects on dendritic spines (Fig. 6G). Compared to controls, PUS7 knockdown significantly reduced dendritic spine density in ILPFC neurons (t-test, *p* < 0.05; Fig. 6H). This structural impairment correlated with fear extinction deficits, demonstrating the essential role of PUS7 in maintaining synaptic integrity during extinction memory formation. These results suggest that PUS7 promotes extinction memory formation at the synaptic level by increasing pseudouridine enrichment on synapse-related genes, thereby facilitating their function and modulating dendritic spine number.

## Discussion

Fear extinction is widely regarded as the neurobiological foundation of exposure-based therapy for PTSD, critically dependent upon the precise regulation of synaptic related functions within the ILPFC [31, 32]. While extensive research has systematically cataloged the roles of transcription factors, non-coding RNAs, and diverse epigenetic mechanisms in various forms of memory [33, 34], the functional contributions of RNA modifications dynamic and reversible regulators of gene expression, remain largely unexplored. This represents a critical gap in the molecular understanding of experience-dependent neuronal plasticity. To date, over 150 chemically distinct RNA modifications have been identified [35], yet only N6-methyladenosine (m^6^A) has been experimentally confirmed to regulate fear extinction [33, 36]. This highlights our limited but encouraging understanding of the diversity and regulatory complexity encoded by RNA modifications in neural circuits. With the advent of advanced epitranscriptomic analysis, pseudouridine, the most abundant RNA modification during development [37], which has recently emerged as a compelling molecular target for elucidating the synaptic substrates of fear extinction. While aberrant Ψ modifications have been implicated in various neurodevelopmental disorders [18], the precise role of Ψ in shaping synaptic function within mature, behaviorally relevant neural circuits remains to be fully elucidated. Here, we focus on the pivotal pseudouridine synthase PUS7 to explore its previously uncharacterized role in learning and memory. PUS7 is known to catalyze the formation of Ψ on tRNAs [25], including the generation of Ψ-tRNA fragments involved in the regulation of protein synthesis in embryonic stem cells [38], as well as the pseudouridylation of select mRNA substrates [39]. This activity suggests that PUS7 may regulate gene expression at multiple levels, in a manner similar to METTL3’s multilayered m^6^A-mediated Gene regulatory mechanisms [40–42], to precisely modulate complex neural networks.

On this conceptual basis, and in line with the established requirement for rapid, activity-dependent transcriptomic reprogramming during fear extinction memory consolidation [34], we turned our attention to RNA modifications as potential mediators of this process. Our findings reveal several key advances: (1) We provide the first evidence that site-specific pseudouridylation catalyzed by PUS7 is closely associated with the stabilization of fear extinction memory in the ILPFC, uncovering a previously unrecognized regulatory mechanism of Ψ modification in memory. (2) Our data demonstrate that PUS7 exerts a critical regulatory role in fear extinction memory, with both behavioral and enzymatic specificity. This provides experimental evidence for the functional specialization of Ψ modification mediated by PUS7. (3) We further elucidate that site-specific Ψ modification dynamically fine-tunes synaptic transcripts, thereby precisely regulating memory formation at the molecular level. This highlights the pivotal role of the PUS7/Ψ axis in governing the regulation and stabilization of fear extinction. Collectively, these findings not only expand our understanding of the molecular mechanisms underlying memory processing, but also suggest new avenues for interventions targeting RNA modifications in the context of fear-related disorders.

Further analysis of potential downstream targets indicated that PUS7 modulates a network of synaptic genes, including Git1, which is a master regulator of dendritic spine morphogenesis [43, 44], Bsn, which serves as the molecular scaffold for presynaptic active zones [45], and Dlgap1, a key regulator of PSD-95 signaling complexes [46]. Through the regulation of this gene network, PUS7 provides an extensive molecular foundation for experience-dependent adaptation and memory formation. These results delineate a mechanistic pathway connecting RNA modification, synaptic gene expression, and ultimately behavioral output.

However, several notable challenges and limitations persist. Although our study identified over 650 potential sites of Ψ modification catalyzed by PUS7, direct evidence linking individual modification events to specific changes in transcript stability or translation remains to be fully established and warrants further experimental validation [47, 48].

While our study provides valuable insights into PUS7 regulation during extinction, there are certain aspects where our approach remains insufficient to fully capture the underlying mechanisms. For example, Our study shows that alterations in mRNA levels are not always matched by corresponding changes in protein abundance. For example, after 10 CS extinction sessions, PUS7 mRNA levels increased, whereas PUS7 protein levels remained elevated for a longer duration. This indicates that mechanisms such as enhanced translation or increased protein stability may sustain PUS7 protein levels even after mRNA expression returns to baseline. These findings highlight the importance of examining both transcript and protein profiles to gain a comprehensive understanding of PUS7 regulation during extinction.We further observed that PUS7 knockdown led to changes in dendritic spine density under baseline conditions, suggesting a constitutive role for PUS7 in maintaining neuronal structure. The dynamic expression of PUS7 during extinction raises the possibility that it contributes to both baseline neural maintenance and experience-dependent adaptations. To further clarify these roles, future studies focused on downstream effector genes and their involvement in extinction memory may provide additional mechanistic insights.With regard to the experimental conditions, EXT mice were repeatedly exposed to the conditioned stimulus, while RC mice did not receive the CS. This distinction introduces additional factors, such as memory retrieval or arousal, that may contribute to the observed differences between groups. Incorporating a variety of control groups in future research will help specify the particular influence of extinction training on molecular and cellular changes.Overall, our data support a specific and evolutionarily conserved role for PUS7 in pseudouridylation within the ILPFC and demonstrate its involvement in the regulation of fear extinction memory. Notably, PUS7 knockdown produced significant changes in baseline pseudouridylation, further indicating its key role in maintaining RNA modification homeostasis. Future work to identify specific targets of PUS7 and to define their functional impact on extinction processes will expand our understanding of how PUS7-mediated modifications shape neural function and behavior.

Our findings demonstrate that site-specific pseudouridylation modifications catalyzed by PUS7-serves as a pivotal molecular mechanism underlying fear extinction. By elucidating spatial parameters of Ψ accumulation within the ILPFC, the enzymatic specificity of PUS7, and the behavioral selectivity related to fear extinction, our study reinforces the concept of the RNA exon as a precise molecular addressing system that could orchestrate adaptive learning and memory. Looking ahead, it will be essential to determine whether pharmacological or genetic modulation of the PUS7-pseudouridylation pathway can enhance the efficacy or specificity of exposure-based interventions for PTSD. Such endeavors are expected to translate these molecular discoveries into more precise and effective therapeutic strategies, thereby highlighting the critical role of RNA modification dynamics in regulating neurobiological processes and behavior.

## Supplementary Information

Below is the link to the electronic supplementary material.

Supplementary Material 1: Fig. S1: Stable expression of pseudouridine synthase mRNAs in the ILPFC across fear extinction; Fig. S2: Open field test in animals treated with PUS7 shRNA; FigS3: mRNA expression of synapse-associated genes following EXT with PUS7 shRNA. Table S1: The primers used in this study. Table S2: Quantitative Profiling of Ψ Distribution in the ILPFC by LC-MS. Table S3: Comparative Analysis of Ψ-Modified Peaks Between EXT and RC Groups. Table S4: PUS7-bound Ψ modification sites were identified through bioinformatic intersection of two datasets: (1) EXT-specific upregulated modifications. (2) EXT-group fRIP-seq PUS7-RNA interactions.

## Data Availability

Data will be made available on request.
