# Supplementary material for: PUS7-dependent Ψ reshapes specific synaptic gene exons to facilitate fear extinction memory formation
Source: Mol Brain. 2025 Oct 15;18:80. doi: 10.1186/s13041-025-01250-6 (PMC12523022; doi:10.1186/s13041-025-01250-6)
Supplement: Supplementary file 1 — Supplementary Material 1: Fig. S1: Stable expression of pseudouridine synthase mRNAs in the ILPFC across fear extinction; Fig. S2: Open field test in animals treated with PUS7 shRNA; FigS3: mRNA expression of synapse-associated genes following EXT with PUS7 shRNA. Table S1: The primers used in this study. Table S2: Quantitative Profiling of Ψ Distribution in the ILPFC by LC-MS. Table S3: Comparative Analysis of Ψ-Modified Peaks Between EXT and RC Groups. Table S4: PUS7-bound Ψ modification sites were identified through bioinformatic intersection of two datasets: (1) EXT-specific upregulated modifications. (2) EXT-group fRIP-seq PUS7-RNA interactions. [file 13041_2025_1250_MOESM1_ESM.zip › Supplementary/Supplementary Table1.docx]

| Primers | (5′-3′) Sequences |
| --- | --- |
| shRNA-Pus7 | CGATTCGAGATTACTCCTTAT |
| Shank1-F1 | CTATTCAGCGGTACCCGGTC |
| Shank1-R1 | TCAGAGAGATCTCCCCCTCG |
| Dlgap1-F1 | CGCTGAGGTCAGTTTCTGGT |
| Dlgap1-R1 | CAAAGGTTCTGATGCCCTGC |
| Agap2-F1 | ATCTGAGTCCCCTGAGTCGG |
| Agap2-R1 | GGCGTGGACAGTTTCTTCCT |
| Dlg4-F1 | GAACCAGCACATCCCTGGAG |
| Dlg4-R1 | GATCTGCAACCTGCCATCCT |
| Rph3a-F1 | GCTCCAGGCAGATGTTGAAG |
| Rph3a-R1 | CCGTGTGTGACGAGGACAAG |
| Bsn-F1 | GGCTGACCCTTCTTCTGCAT |
| Bsn-R1 | CATGACTACGATGAGCCCCC |
| Palm-F1 | GAGAAGCGTAGGAAGCAGGC |
| Palm-R1 | GTACTGCAGCTGTCTTCGGT |
| Phactr1-F1 | CCCATCCGGAGGAGGAGTAA |
| Phactr1-R1 | TGCTGACGTGTGCTTGAACT |
| Epb41l1-F1 | TCGGGAAAGATGTCCTCACC |
| Epb41l1-F2 | GGTGACATGGGTGGTGGTAG |
| Git1-F1 | GGTCACCGCCAAAGACCTC |
| Git1-R1 | CAGCCCCATACACTACCAGC |
| PUS7-CDS-F | atggaaatgacaagcacgtccct |
| PUS7-CDS-R | tcatcgcagccagctcgt |

Table1: The primers used in this study
