# Supplementary material for: PUS7-dependent Ψ reshapes specific synaptic gene exons to facilitate fear extinction memory formation
Source: Mol Brain. 2025 Oct 15;18:80. doi: 10.1186/s13041-025-01250-6 (PMC12523022; doi:10.1186/s13041-025-01250-6)
Supplement: Supplementary file 1 — Supplementary Material 1: Fig. S1: Stable expression of pseudouridine synthase mRNAs in the ILPFC across fear extinction; Fig. S2: Open field test in animals treated with PUS7 shRNA; FigS3: mRNA expression of synapse-associated genes following EXT with PUS7 shRNA. Table S1: The primers used in this study. Table S2: Quantitative Profiling of Ψ Distribution in the ILPFC by LC-MS. Table S3: Comparative Analysis of Ψ-Modified Peaks Between EXT and RC Groups. Table S4: PUS7-bound Ψ modification sites were identified through bioinformatic intersection of two datasets: (1) EXT-specific upregulated modifications. (2) EXT-group fRIP-seq PUS7-RNA interactions. [file 13041_2025_1250_MOESM1_ESM.zip › Supplementary/Supplementary Figures.docx]

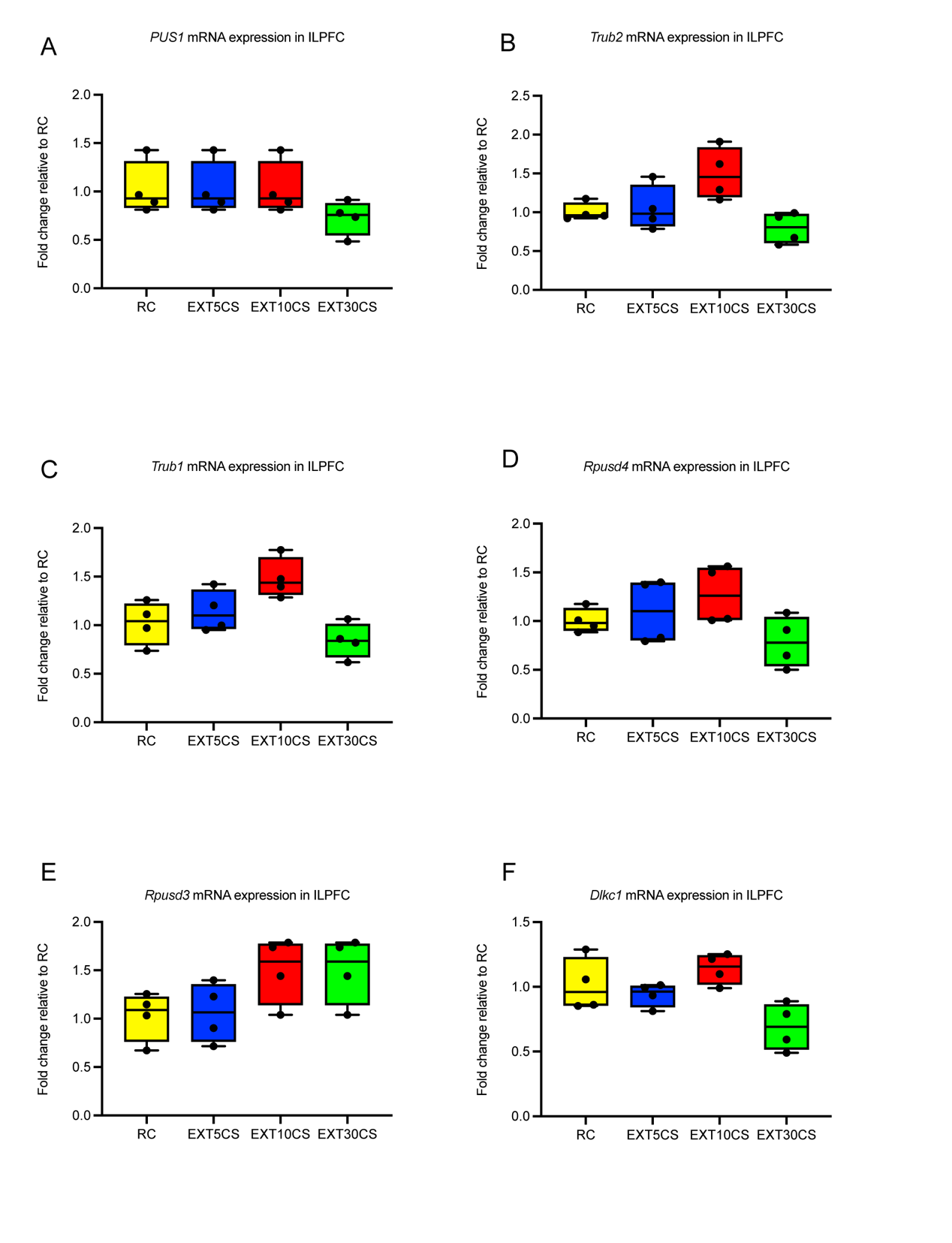


**Fig. S1** Expression of pseudouridine synthase mRNAs in the ILPFC across fear extinction process. **(A-E)** RT-qPCR analysis revealed no significant differences in the mRNA expression levels of key pseudouridine (Ψ) synthase enzymes in the ILPFC during 5CS. 10CS and 30CS extinction conditions, respectively. Analyzed enzymes include: **(A)** PUS1, **(B)** Tru2, **(C)** Trub1, **(D)** Rpusd4, **(E)** Rpusd3, and **(F)** Dlkc1(All panels: One-way ANOVA. *p* > 0.05; n = 4 biological replicates per group)


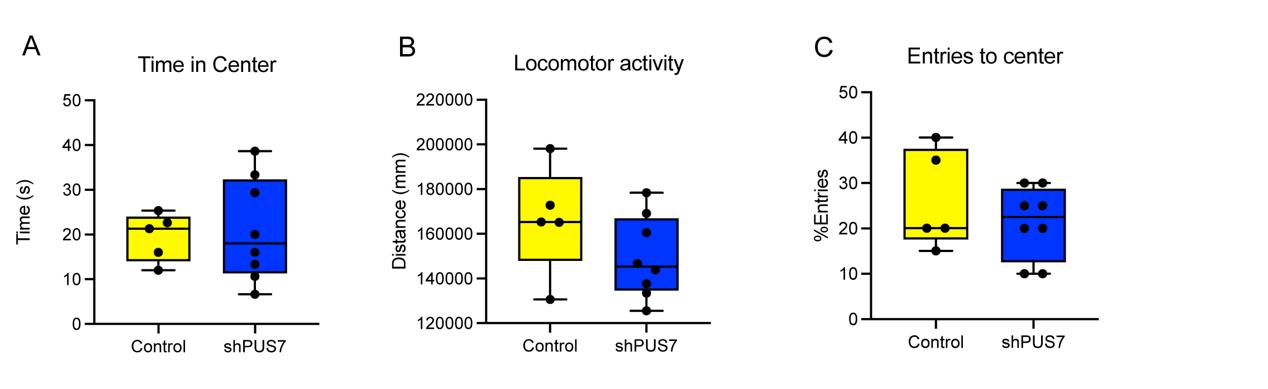


**Fig. S2** Open field test in animals treated with PUS7 shRNA. **(A)** Time spent in the central zone (*p* > 0.05). **(B)** Total locomotor activity measured as distance traveled (*p* > 0.05). **(C)** Number of entries into the central zone (two-tailed unpaired Student’s *t* test; *p* > 0.05; n = 5-8 biological replicates per group)


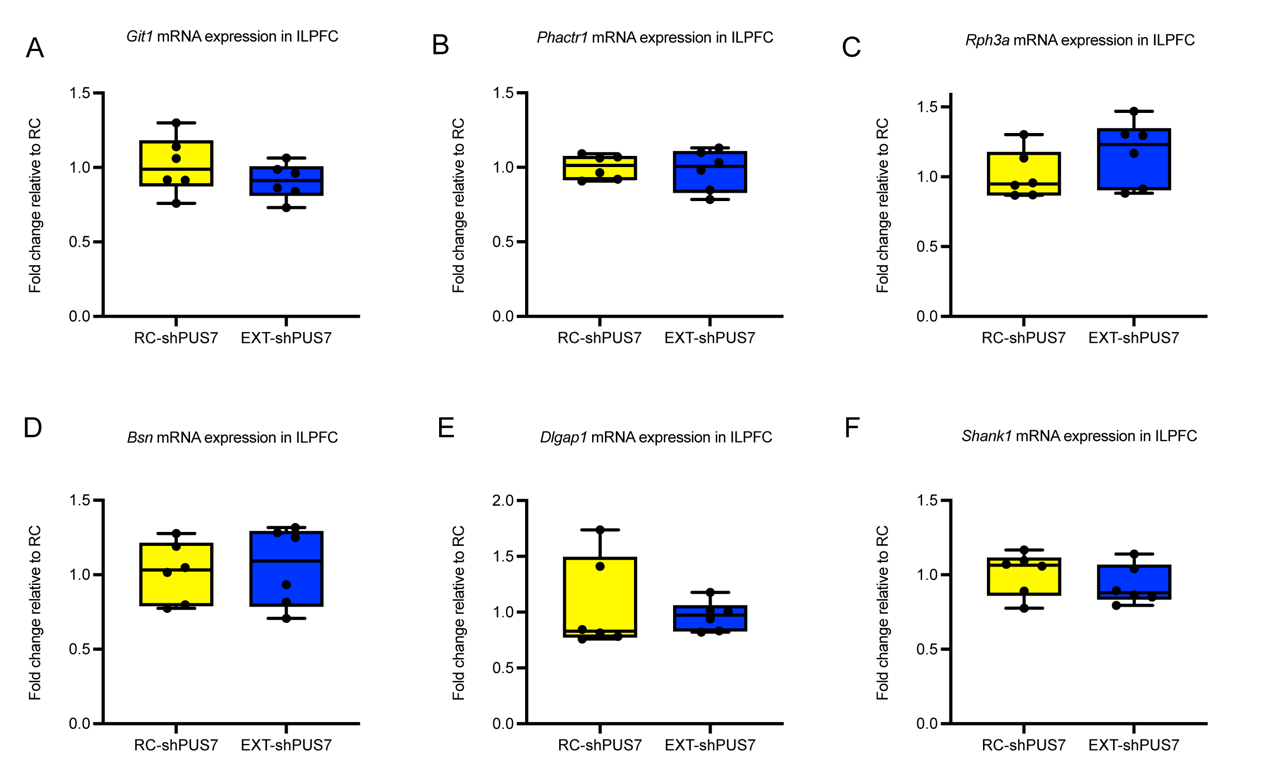


**Fig. S3.** mRNA expression of synapse-associated genes following EXT with PUS7 shRNA. quantified by RT-qPCR. **(A)** Git1, **(B)** Phactr1, **(C) R**ph3a, **(D)** Bsn, **(E)** Dlgap1, and **(F)** Shank1(two-tailed unpaired Student’s t test). Error bars represent SEM; p > 0.05. n = 6 biological replicates per group).
