# Supplementary figures and images for: PUS7-dependent Ψ reshapes specific synaptic gene exons to facilitate fear extinction memory formation
Source: Mol Brain. 2025 Oct 15;18:80. doi: 10.1186/s13041-025-01250-6 (PMC12523022; doi:10.1186/s13041-025-01250-6)

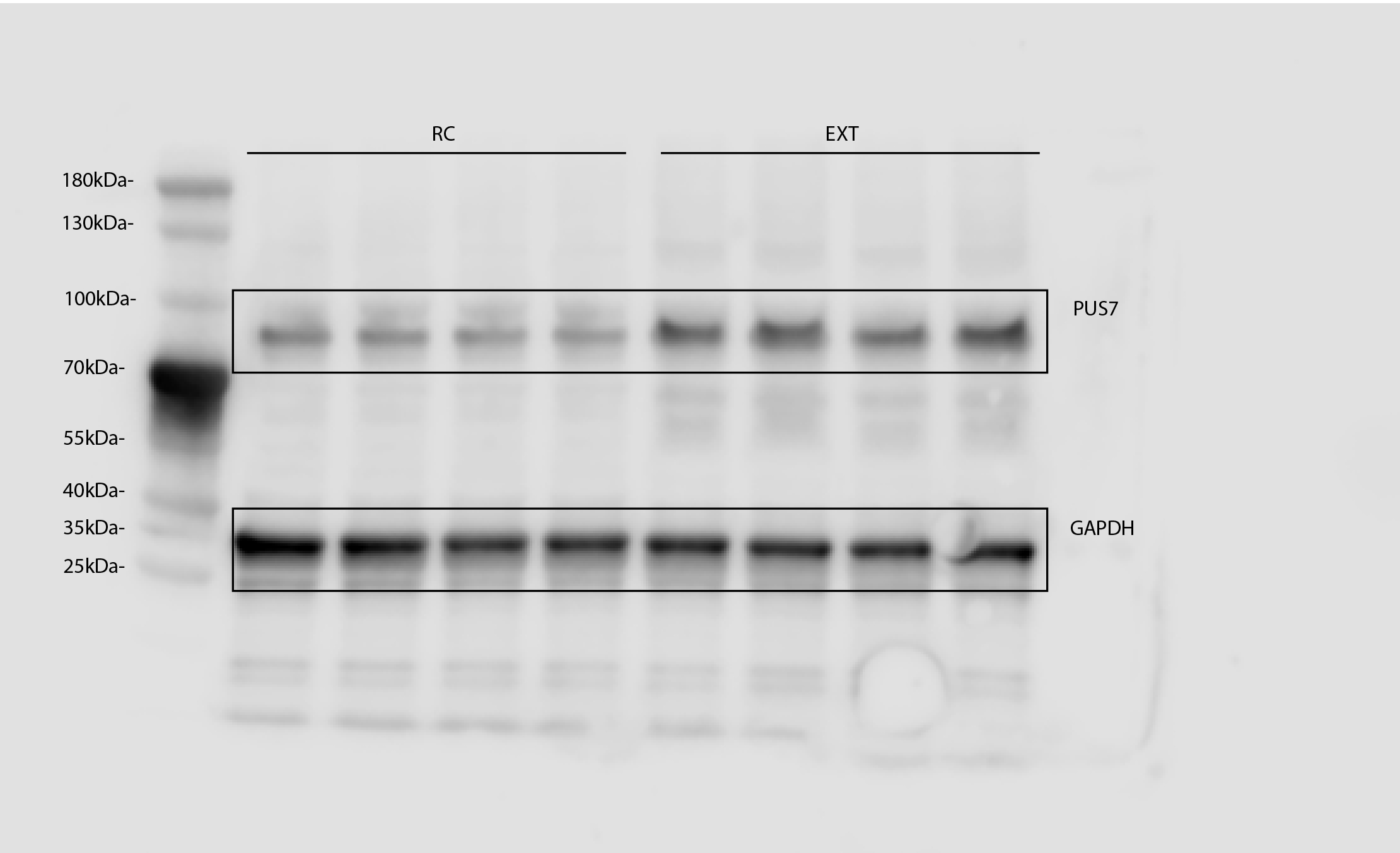

Supplement: Supplementary file 1 — Supplementary Material 1: Fig. S1: Stable expression of pseudouridine synthase mRNAs in the ILPFC across fear extinction; Fig. S2: Open field test in animals treated with PUS7 shRNA; FigS3: mRNA expression of synapse-associated genes following EXT with PUS7 shRNA. Table S1: The primers used in this study. Table S2: Quantitative Profiling of Ψ Distribution in the ILPFC by LC-MS. Table S3: Comparative Analysis of Ψ-Modified Peaks Between EXT and RC Groups. Table S4: PUS7-bound Ψ modification sites were identified through bioinformatic intersection of two datasets: (1) EXT-specific upregulated modifications. (2) EXT-group fRIP-seq PUS7-RNA interactions. [file 13041_2025_1250_MOESM1_ESM.zip › Supplementary/full uncropped Gel Blots image.jpg]
